# Identifying Leprosy and Those at Risk of Developing Leprosy by Detection of Antibodies against LID-1 and LID-NDO

**DOI:** 10.1371/journal.pntd.0004934

**Published:** 2016-09-22

**Authors:** Francianne M. Amorim, Maurício L. Nobre, Leonardo C. Ferreira, Larissa S. Nascimento, Alesson M. Miranda, Glória R. G. Monteiro, Kathryn M. Dupnik, Malcolm S. Duthie, Steven G. Reed, Selma M. B. Jeronimo

**Affiliations:** 1 Department of Biochemistry, Bioscience Center, and Institute of Tropical Medicine of Rio Grande do Norte, Universidade Federal do Rio Grande do Norte, Natal, Rio Grande do Norte, Brazil; 2 Post-graduate Program in Tropical Medicine, Instituto Oswaldo Cruz, Fiocruz, Rio de Janeiro, Brazil; 3 Hospital Giselda Trigueiro, Rio Grande do Norte Health Secretariat, Natal, Rio Grande do Norte, Brazil; 4 Center for Global Health, Weill Cornell Medical College, New York, New York, United States of America; 5 Infectious Disease Research Institute, Seattle, Washington, United States of America; 6 Instituto Nacional de Ciência e Tecnologia de Doenças Tropicais (INCT-DT), Salvador, Bahia, Brazil; University of Tennessee, UNITED STATES

## Abstract

Leprosy is caused by *Mycobacterium leprae* infection and remains a major public health problem in many areas of the world. Challenges to its timely diagnosis result in delay in treatment, which is usually associated with severe disability. Although phenolic glycolipid (PGL)-I has been reported as auxiliary diagnostic tool, currently there is no serological assay routinely used in leprosy diagnosis. The aim of this study was to evaluate the effectiveness of two related reagents, LID-1 and LID-NDO, for the detection of *M*. *leprae* infection. Sera from 98 leprosy patients, 365 household contacts (HHC) and 98 endemic controls from Rio Grande do Norte, Brazil, were evaluated. A subgroup of the HHC living in a hyperendemic area was followed for 7–10 years. Antigen-specific antibody responses were highest in multibacillary (MB) at the lepromatous pole (LL/BL) and lowest in paucibacillary (PB) at the tuberculoid pole (TT/BT). A positive correlation for both anti-LID-1 and anti-LID-NDO antibodies was found with bacterial burden (LID-1, r = 0.84, *p*<0.001; LID-NDO, r = 0.82, *p*<0.001), with higher sensitivity than bacilloscopy. According to *Receiver Operating Curve*, LID-1 and LID-NDO performed similarly. The sensitivity for MB cases was 89% for LID-1 and 95% for LID-NDO; the specificity was 96% for LID-1 and 88% for LID-NDO. Of the 332 HHC that were followed, 12 (3.6%) were diagnosed with leprosy in a median time of 31 (3–79) months after recruitment. A linear generalized model using LID-1 or LID-NDO as a predictor estimated that 8.3% and 10.4% of the HHC would become a leprosy case, respectively. Together, our findings support a role for the LID-1 and LID-NDO antigens in diagnosing MB leprosy and identifying people at greater risk of developing clinical disease. These assays have the potential to improve the diagnostic capacity at local health centers and aid development of strategies for the eventual control and elimination of leprosy from endemic areas.

## Author Summary

Despite the substantial decrease in its prevalence, leprosy continues to be a worldwide challenge. Early diagnosis and treatment are important to interrupt transmission. Currently, there is no gold standard for the diagnosis of leprosy. Bacilloscopy and histopathology studies are complementary exams that provide high specificity but low sensitivity. It is therefore important to seek alternative tools to achieve rapid and accurate diagnosis. The state of Rio Grande do Norte, in Brazil, has municipalities’ that are considered hyperendemic for leprosy, such as Mossoró, the one included in this study. This city presented an average of new case detection rate (NCDR) of 45.4/100.000 inhabitants per year from 2001 to 2013, much higher than Brazil’s NCDR, which is currently 15.3. Here, we show that the utility of the recombinant antigens LID-1 and LID-NDO to diagnose MB patients and detect asymptomatic *M*. *leprae* infection. In addition, we showed that antibody levels were related to the clinical form of leprosy as well as to the bacillary load. Interestingly, we observed that serum levels of LID-1/LID-NDO antibodies can be used to predict leprosy development among HHC. The assays have the potential to eventually be implemented as point of care at local health centers.

## Introduction

Leprosy is caused by *Mycobacterium leprae* infection and, despite the availability of free, effective multidrug therapy (MDT), it remains a major public health problem. Leprosy is the leading worldwide cause of non-traumatic peripheral neuropathy. Challenges to timely diagnosis result in delay in treatment, which leads to severe disability [1]. Of importance, one third of the leprosy cases develop immunopathologic reactions, which tend to be an additional cause of disability [2–4]. India and Brazil are the two countries with the largest number of cases [5].

Infection with *M*. *leprae* can evolve into a wide range of outcomes, from asymptomatic infection to disseminated disease. Presentation of clinical leprosy is also on a spectrum, varying between tuberculoid and lepromatous poles [6]. The tuberculoid pole (TT) is characterized by few, well-defined, hypopigmented, hypoesthetic lesions. Histopathologic analysis of lesions of TT patients usually has few or no bacilli [7]. In contrast, patients at the lepromatous pole (LL) present with numerous skin lesions, infiltration of skin and sometimes internal organs. Histology reveals foamy macrophages containing large numbers of bacilli within a disorganized lymphocytic infiltrate. Between these two poles, there are intermediate clinical forms as borderline-tuberculoid (BT), borderline-borderline (BB) and borderline-lepromatous (BL) [6]. People with TT leprosy present a strong Th1 cell mediated immune response and are usually seronegative for anti-*M*. *leprae* antibodies, while people with LL leprosy skew toward a Th2 pole and a strong antibody-mediated responses that do not control bacilli replication and are usually seropositive [8;9].

The diagnosis of leprosy is based on clinical examination, bacilloscopy and histopathology. Although, bacilloscopy and histopathology provide a high specificity, they have low sensitivity [10]. These tests also present technical and practical limitations because of their invasive nature, required materials, and need for specific technical expertise. The World Health Organization (WHO) developed a simpler classification to be applied in areas that lack the ability to carry on histopathological studies. Under WHO guidelines, patients are classified as paucibacillary (PB) when presenting with up to five lesions, and as multibacillary (MB) if they present with more than five lesions [11–13]. It is important to seek alternative and practical tools that can help to achieve the earliest possible diagnosis and therapy to interrupt both disease development and *M*. *leprae* transmission.

Serological tests can be used following finger-prick blood collection to evaluate antibody responses to *M*. *leprae* specific antigens. Phenolic glycolipid (PGL)-I has been used as the antigen [14–16]. Patients who have multibacillary leprosy produce large amounts of IgM directed towards PGL-I. The magnitude of anti-PGL-I IgM correlates well with the bacillary load [17]. Serologic responses are also detected against many proteins. The recombinant protein antigens ML0405 and ML2331 have exhibited high sensitivity for the detection of leprosy remove throughout the clinical spectrum when tested against large panels of sera from many different geographic regions (Philippines, Brazil and Japan) [18–20]. A fusion protein, LID-1 (leprosy IDRI diagnostic-1) was developed by fusing the *ml0405* and *ml2331* genes to produce a single chimeric protein with a better sensitivity than the original proteins alone [21;22]. Recently, LID-1 and PGL-I epitopes were conjugated to form LID-NDO. Prospective studies using LID-NDO showed high sensitivity and specificity [23].

The sensitivity of serological tests for leprosy varies depending on the geographic origin of the sera; therefore, response profile to a particular antigen needs to be evaluated in diverse populations [24]. As such, the primary objective of this study was to evaluate the ability of LID-1 and LID-NDO to discriminate the different clinical forms of leprosy in the state of Rio Grande do Norte, Brazil. We also evaluated whether positive responses against these antigens could provide a predictive value for leprosy development in household contacts of clinically identified leprosy cases.

## Methods

### Study design and population

**Fig 1:**
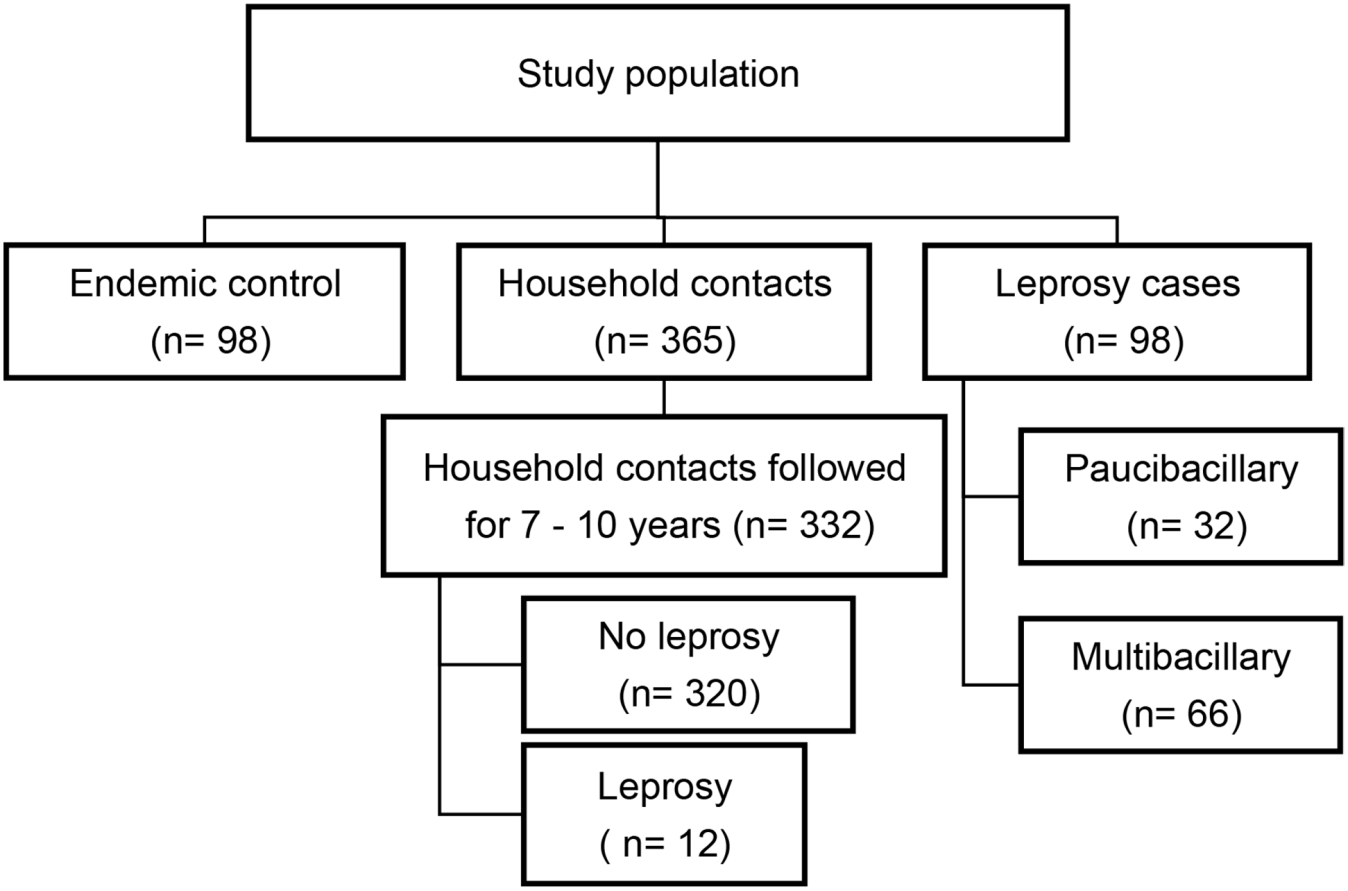
Population from Rio Grande do Norte-Brazil tested for LID-1 and LID-NDO. Analysis of LID-1 and LID-NDO ELISA as diagnostic tools to identify leprosy patients, to detect asymptomatic infection and to predict leprosy development.

Initially, to evaluate the ability of LID-1 and LID-NDO to detect the different clinical forms of leprosy and asymptomatic infection, we used samples collected at diagnosis and household contacts from different areas in the State of Rio Grande do Norte (Fig 1).

A total of 98 cases of leprosy were recruited from two leprosy referral centers in the state of Rio Grande do Norte, Brazil, between 2005–2014. Patients were recruited at Hospital Rafael Fernandes, Mossoró, and Hospital Giselda Trigueiro, Natal. According to the State of Rio Grande do Norte Health Secretariat database, Mossoró and Natal are the two municipalities with the largest number of leprosy cases in the state of Rio Grande do Norte [25;26]. Cases of leprosy were diagnosed as PB or MB by standard criteria and blood samples were collected prior to the initiation of MDT [11]. A subgroup of those patients (n = 50) which there were available biopsy results were characterized in accordance to Ridley and Jopling classifications [6].

Healthy household contacts (HHC) were recruited, as described in Moura *et al* (2013) [27]. Serum samples of these HHC were studied to assess the utility of serological assays to predict disease development. A total of 365 household contacts were clinically screened for leprosy, of whom 183 were contacts of PB and 167 were MB contacts. Serum samples from people residing in the endemic area, but with no history of contact with a leprosy case was used as endemic control (EC, n = 98). Epidemiological data for the groups are presented in Table 1. The mean age of leprosy cases was 45.6 years, 52% were female and 67.3% of the cases were MB. A subgroup of the HHC (n = 332) living in the hyperendemic area of Mossoró (recruited from 2006 to 2008) were followed for 7–10 years (Fig 1). In this cohort, through the analysis of Rio Grande do Norte leprosy database, it was identified those HHC that became a leprosy case in this interval of time.

**Table 1:** Characterization of the study population.

Variable	EC (n = 98)	HHC (n = 365)	Leprosy (n = 98)	p-value
Age, years	33.8 (±11)	32.7 (±20)	45.6 (±16)	<0.001^b^^,^^c^
Sex, n (%)				
Male	50 (51.5)	144 (39.6)	51 (52.0)	<0.05^a^^,^^c^
Female	47 (48.5)	221 (60.4)	47 (48.0)	
City				
Natal	62 (64.6)	12 (3.3)	13 (13.3)	<0.001^a^^,^^b^^,^^c^
Mossoró	0 (0)	332 (90.9)	50 (51.0)	
Others	34 (35.4)	21 (5.8)	35 (35.7)	
Cases, n (%)				
PB	-	-	32 (32.7)	-
MB	-	-	66 (67.3)	
Household contacts from				
PB	-	183 (52.3)	-	-
MB	-	167 (47.7)	-	

Age shown as mean (±sd). Sex and age were compared through chi-squared and Tukey’s test, respectively. (EC: endemic control; HHC: household contacts of leprosy patients; PB: paucibacillary; MB: multibacillary).

^a^ EC vs HHC.

^b^ EC vs Leprosy.

^c^ HHC vs Leprosy.

### Antibody detection

Antibodies to LID-1 and LID-NDO were detected by enzyme linked immunoassay (ELISA), following previously established protocol [28]. The optical density (OD) for each sample in antigen-specific ELISA was obtained after subtracting the OD reading obtained in the placebo plate. Two control samples were included in each plate to normalize the overall data and account for inter-plate variations.

### Ethical considerations

The protocol was assessed and approved by the Universidade Federal do Rio Grande do Norte Ethical Committee (CEP-UFRN) and by the Brazilian National Ethical Committee (CONEP/CNS/ Ministério da Saúde, Brasília). All participants or their legal guardians signed informed consent forms prior to sample collection.

### Statistical analyses

We used analysis of variance (ANOVA) with the *post-hoc* Tukey’s test to evaluate mean differences between groups, assuming that OD values were normally distributed. The cut-offs used for sensitivity and specificity calculations were based on optimized OD thresholds generated by Receiver Operating Characteristic (ROC) analysis, assuming three different scenarios for the use of the antigens, which included: 1. The diagnose of multibacillary leprosy in a general endemic population; 2. The diagnose of multibacillary in a high risk population and 3. To identify asymptomatic *M*. *leprae* infected subjects. The diagnostic performances of LID-1 and LID-NDO tests were assessed by comparison of the Area Under the Curve (AUC) with DeLong’s test. We used the longitudinal setting (Fig 1) to estimate the predicted probability of a household contact (HHC) to develop leprosy, through simple logistic regression model using OD values as predictor variable. Additionally, we ran multiple logistic regression adjusting for age and sex, but no confounding effects were detected. All statistical analyses were performed in R, assuming a significance level of 0.05.

## Results

### Anti-LID-1 and LID-NDO antibody levels

**Fig 2:**
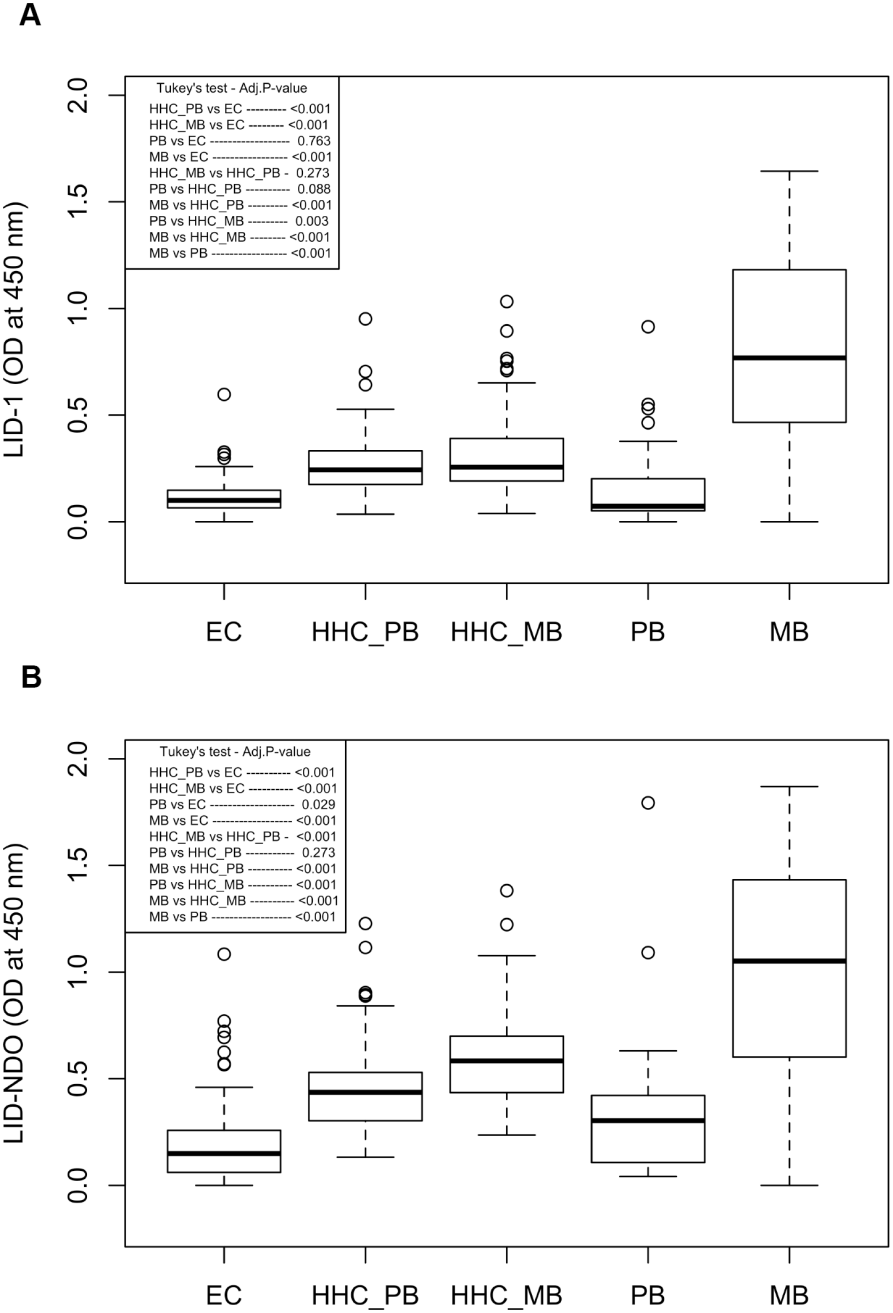
Comparison of specific antibody responses among endemic controls, household contacts and leprosy cases. The optical density (OD) for each sample in antigen-specific ELISA was obtained after subtracting the OD reading obtained with a placebo. A) LID-1 results. B) LID-NDO results. (EC: endemic control; HHC_PB: household contacts of paucibacillary patients; HHC_MB: household contacts of multibacillary patients; PB: paucibacillary; MB: multibacillary).

**Fig 3:**
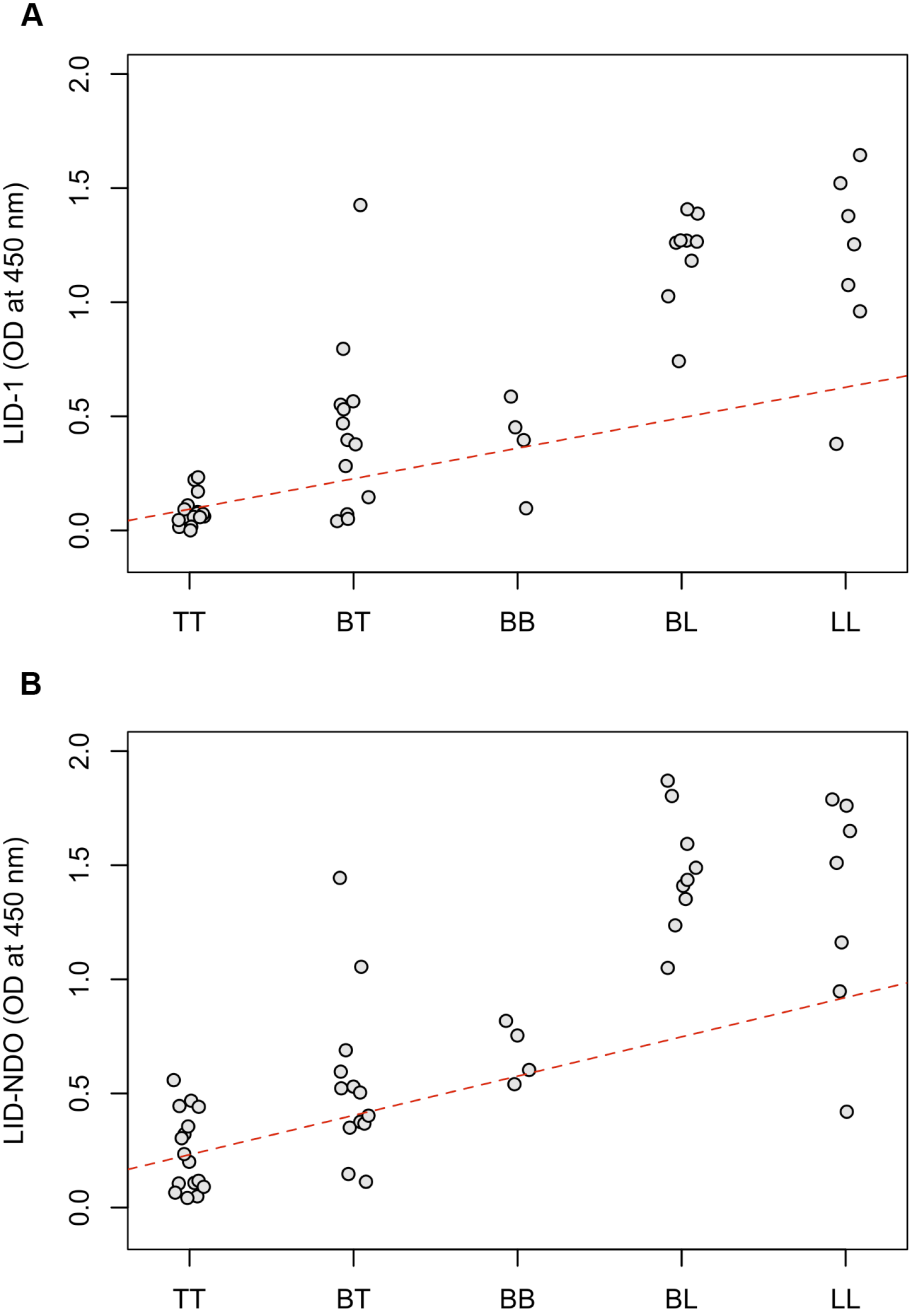
Linear increase of antibody levels across the leprosy spectrum. Sera from leprosy patients characterized as TT (tuberculoid), BT (borderline-tuberculoid), BB (borderline-borderline), BL (borderline-lepromatous) or LL (lepromatous) were analyzed for antibodies against LID-1 and LID-NDO. A) The slope of the fitted line (red) suggests an increment of 0.299 on the LID-1 mean, as group moves from TT to LL poles (p<0.001). B) The slope of the fitted line (red) suggests an increment of 0.319 on the LID-NDO mean, as group moves from TT to LL poles (p<0.001).

We evaluated the specific antibody profile to *M*. *leprae* antigens for leprosy cases, household contacts and endemic controls (Fig 1). The magnitude of the antibody responses varied according to the operational classification of patients (as either PB or MB), (Fig 2). For both antigens, MB patients presented higher levels of antibodies compared to all other groups (Fig 2A and 2B). PB patients presented low levels of antibodies, which was not different from those presented by EC or HHC of PB patients. As expected, HHC of MB patients presented a mean OD higher than that observed in HHC of PB patients, although this difference was only significant when LID-NDO was used (Fig 2B). MB patients showed a great variability in the levels of specific antibodies, which was readily apparent when samples were stratified by Ridley and Jopling classification (Fig 3A). By applying a polynomial model, we detected a linear increase of antibody levels across the spectrum from TT to LL, for both LID-1 and LID-NDO (Fig 3A and 3B). The comparison of LID-1 and LID-NDO mean OD between the different clinical forms of leprosy is presented in S1 and S2 Tables, respectively. There was an increment of 0.299 on the LID-1 mean from TT to LL poles (p < 0.001) (Fig 3A). For LID-NDO, this increment was of 0.319 (p<0.001) (Fig 3B).

### LID-1 and LID-NDO ELISA as diagnostic tools

**Fig 4:**
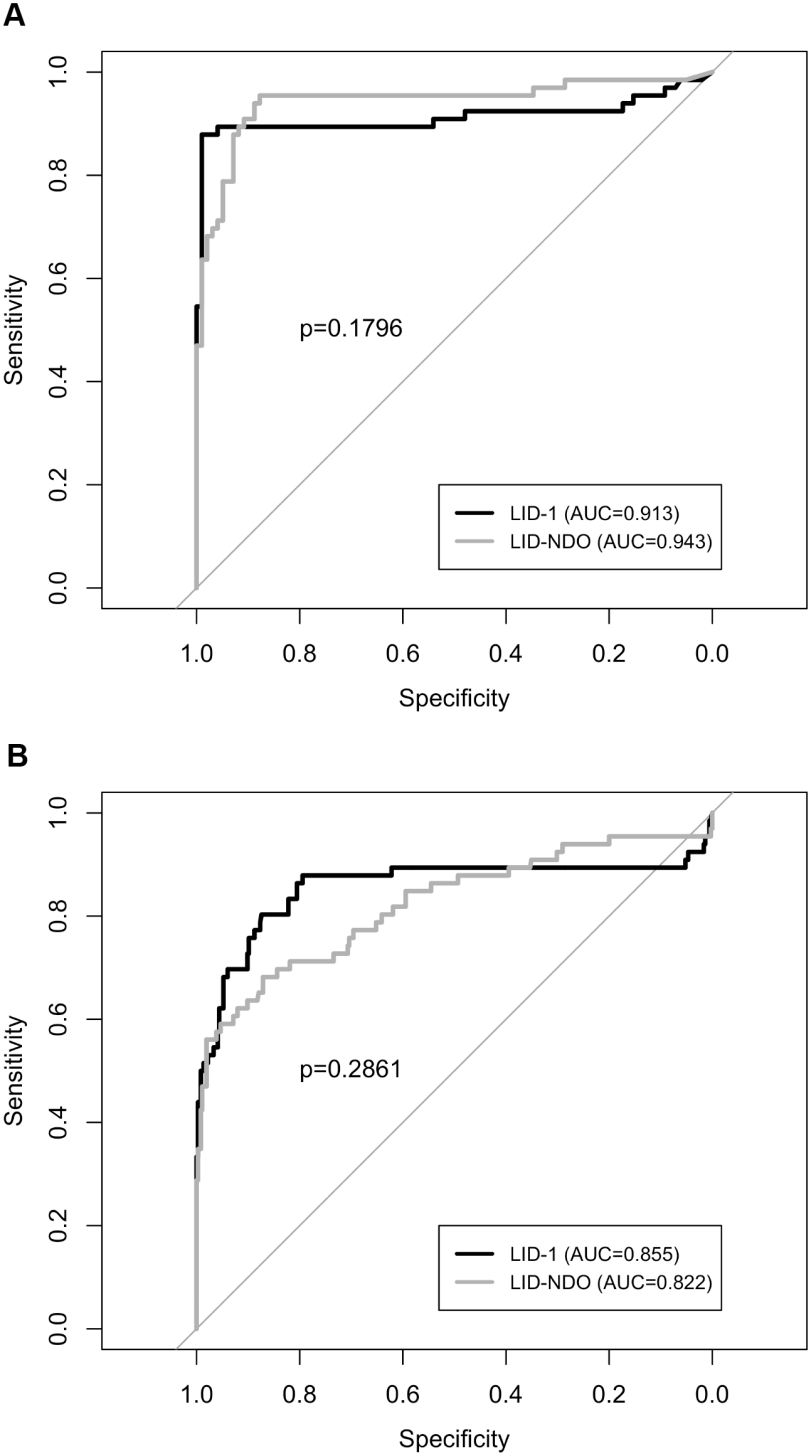
Receiver Operating Characteristic (ROC) curves comparing the ability of LID-1 and LID-NDO to identify MB in endemic general population (A) and in a high-risk population (B). A total of 98 endemic controls (A) and 365 household contacts (B) were used in the analysis along with 66 MB cases. There was no significant difference between the two tests as demonstrated by the area under the curve (AUC).

To compare the ability of the two recombinant antigens to diagnose MB leprosy in ELISA assay and to establish a threshold for positive responses we compared data in both a 2x2 table which had the mean OD of the endemic control plus three times the standard deviation was used (S3 Table) and the ROC curve (Table 2 and Fig 4). The use of a 2x2 table showed that despite a high specificity obtained for both antigens, a relatively low sensitivity was found, especially when considering PB cases (either PB alone or combined with MB) (S3 Table). Therefore, we used ROC curves to determine the optimal threshold for identification of positive samples to compare the performance of the two recombinant antigens. Our analysis considered three different applicability for the ELISA to: 1. To diagnose MB cases in a general endemic population; 2. To diagnose MB cases in a high risk population (*e*.*g*. household contacts); and 3. To identify asymptomatic-infected individuals (Table 2). The sensitivity of ELISA to diagnose MB cases in the endemic population was 89% for LID-1 and 95% for LID-NDO; the specificity was 96% for LID-1 and 88% for LID-NDO.

**Table 2:** Parameters estimated through ROC analysis with regard to three distinct scenarios.

		LID-1			LID-NDO		
Scenario	Comparison	Cut-off	Sens	Spec	Cut-off	Sens	Spec
1. Detect MB cases in a general endemic population	EC vs MB	0.276	0.894	0.959	0.332	0.954	0.876
2. Detect MB cases in a high risk population (*e*.*g*. household contacts)	HHC vs MB	0.423	0.803	0.874	0.688	0.712	0.819
3. Identify asymptomatic-infected individuals	EC vs HHC	0.156	0.816	0.806	0.288	0.871	0.827

Sen: sensitivity; Spec: specificity.

The ROC curves used to compare the ability of LID-1 and LID-NDO ELISA to detect MB patients in either the general population or a high-risk population are presented in Fig 4. The AUC was 0.913 for LID-1 and 0.943 for LID-NDO, with no significant difference between the two antigens (p = 0.1796) to identify MB cases among the general population (Fig 4A). To identify MB cases among HHC, the antigens performance was also similar (p = 0.2861) (Fig 4B).

**Fig 5:**
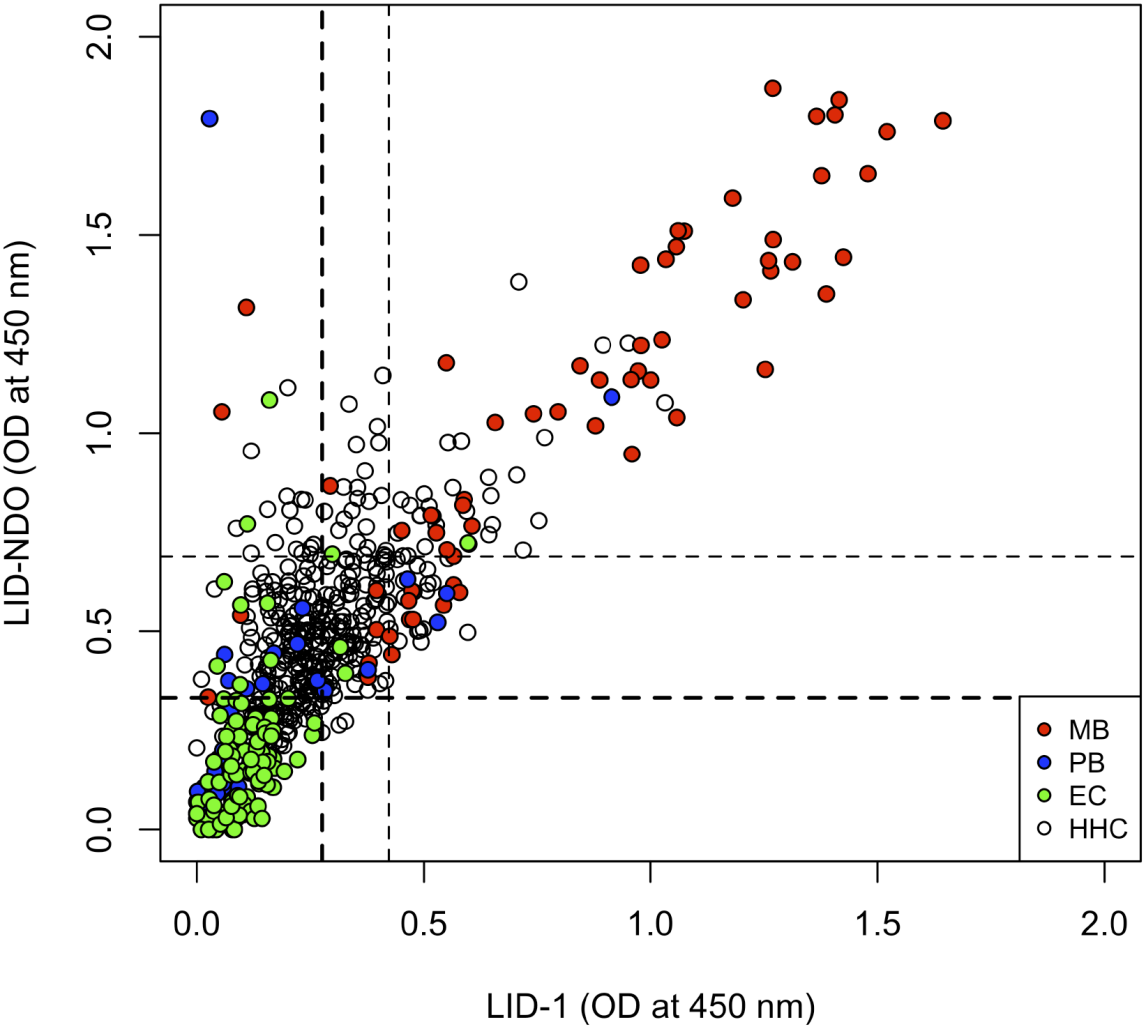
Scatter plot for specific antibody levels and simultaneous tests results. Each sample is distinguished by an individual marker. The Pearson’s coefficient correlation was r = 0.84 (p<0.001). Dashed lines represent cut-offs to diagnose MB cases according to scenarios 1 (darkest lines) and 2 (lightest lines). See Table 2. Net sensitivity for scenario 1 = (0+59+4)/66 = 0.951. Net specificity for scenario 1 = 86/98 = 0.878. Net sensitivity for scenario 2 = (3+44+9)/66 = 0.848. Net specificity for scenario 2 = 336/365 = 0.908. (MB: multibacillary; PB: paucibacillary; EC: endemic control; HHC: household contacts).

The scatter plot in Fig 5 represents the mean OD obtained for each sample with LID-1 and LID-NDO and the cut-offs obtained considering the scenarios 1 and 2. Despite the strong correlation between the levels of anti-LID-1 and anti-LID-NDO (r = 0.84, p<0.001), we observed that some PB patients were negative for LID-1 and positive for LID-NDO. However, all PB patients that were positive for LID-1 were also positive for LID-NDO. Considering scenario 1, the net sensitivity (95%) and net specificity (88%) were similar to those observed with the use of LID-1 or LID-NDO alone. However, considering the scenario 2 the net sensitivity (85%) and specificity (91%) were higher than those observed with the use of only one of the tests.

**Fig 6:**
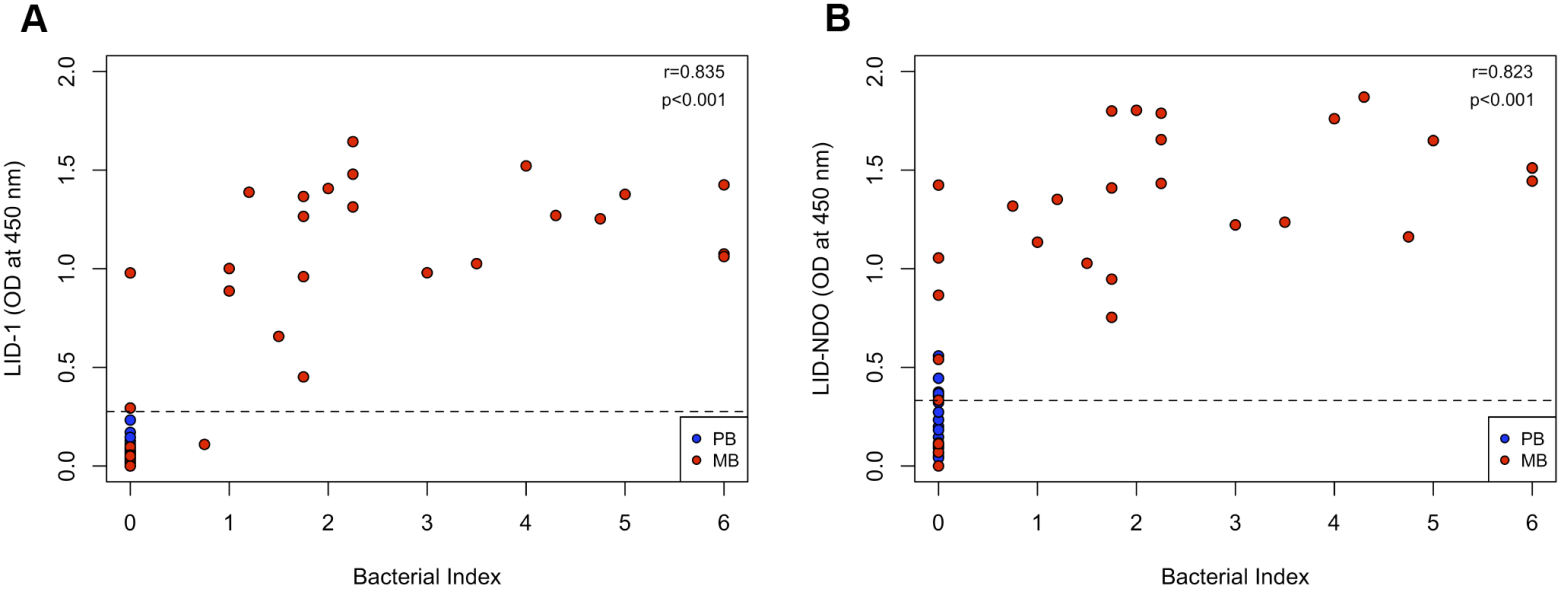
Correlation between ELISA results with bacilli index (BI). Responses against LID-1 (A) and LID-NDO (B) are plotted against the BI (0–6) of leprosy patients. Each sample is distinguished by an individual marker. Strong positive correlations were observed for both anti-LID-1 antibodies (r = 0.84, p<0.001) and anti-LID-NDO (r = 0.82, p<0.001) with the bacterial index. Dashed line represents the cut-off for scenario 1 (0.276 for LID-1 and 0.332 for LID-NDO).

We observed strong positive correlations for both anti-LID-1 (r = 0.84, p<0.001) and anti-LID-NDO (r = 0.82, p<0.001) ELISA data with the bacterial index of leprosy cases (Fig 6A and 6B). However, the ELISA presented with a higher sensitivity than bacilloscopy exam. Among a total of 50 leprosy patients examined, bacilloscopy was negative in 28 cases (*i*.*e*. Bacterial Index = 0). In contrast, LID-NDO ELISA identified 5/20 and 5/8 of these PB and MB cases, respectively. These numbers yield a sensitivity to diagnose leprosy *per se* equal to 44% (22/50) for bacilloscopy but increased to 64% (32/50) for LID-NDO ELISA.

### LID-1 and LID-NDO serology to detect asymptomatic infection among household contacts

**Fig 7:**
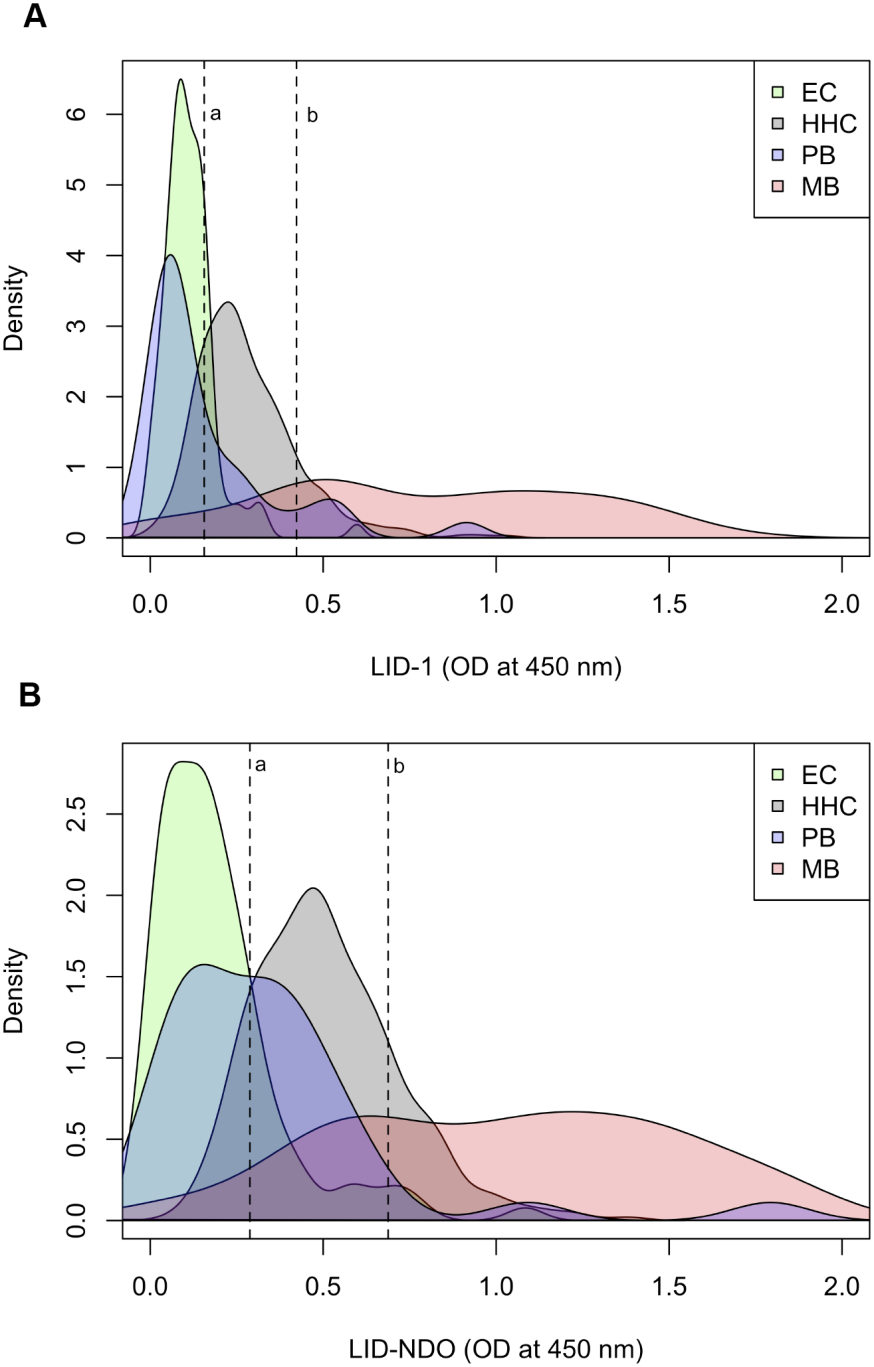
Cut-offs used to define HHC individuals exposed to *M*. *leprae* infection (A) and HHC positive for MB diagnosis (B). Data distribution according to specific antibodies levels. (EC: endemic control; HHC: household contacts; PB: paucibacillary; MB: multibacillary).

It is well documented that HHC of a leprosy case are at higher risk of being exposed to *M*. *leprae* and, consequently, of developing leprosy than the general population. We therefore, considered the distribution of specific antibodies levels, establishing a cut-off to define those HHC exposed to *M*. *leprae* and a cut-off to determine those HHC that presented positive responses equivalent to MB leprosy case. Irrespective of the antigen used, it is apparent that most of the HHC that was recruited in our study were previously exposed to *M*. *leprae* infection (Fig 7A and 7B). We also observed that some of the HHC presented high antibody levels, with OD similar to those measured in sera from MB patients (Fig 7A and 7B).

### Longitudinal study to predict the development of leprosy

To determine if antigen-specific antibody responses could predict disease development, we followed 332 HHC from Mossoró for a minimum of 7 years. Among those HHC, 12 (3.6%) developed leprosy, as reported to the Minister of Health. The diagnosis of leprosy among these HHC occurred at a median of 31 (3–79) months after their recruitment into the study. Among the HHC that developed disease, at the time of recruitment 25% (3/12) presented detectable antibody responses against LID-1 and 33.3% (4/12) had responses against LID-NDO. These positivity percentages were approximately twice the percentages observed for all HHC recruited in the study (LID-1 = 11.9% and LID-NDO = 17.8%). It was observed that 50% (6/12) of the HHC that developed leprosy presented as MB. Interestingly, when we compared the mean OD of HHC that developed disease (LID-1 OD = 0.480 and LID-NDO OD = 0.879) against the mean OD of HHC that did not develop disease (LID-1 OD = 0.267 and LID-NDO OD = 0.492) a significant difference was observed between these groups (p = 0.01 for LID-1 and p = 0.003 for LID-NDO).

In order to evaluate the ability of the serological test to predict disease, we modeled the log odds of the individual being a MB as function of LID-1 and LID-NDO OD values. The predicted probabilities were then calculated from the fitted logit model, as

logodds(Yi=MB)=β0 + β1ODi

$$pi.hat=\frac{e^{\beta 0\;+\;\beta 1ODi}}{1+e^{\beta 0\;+\;\beta 1ODi}}$$ (1)

**Fig 8:**
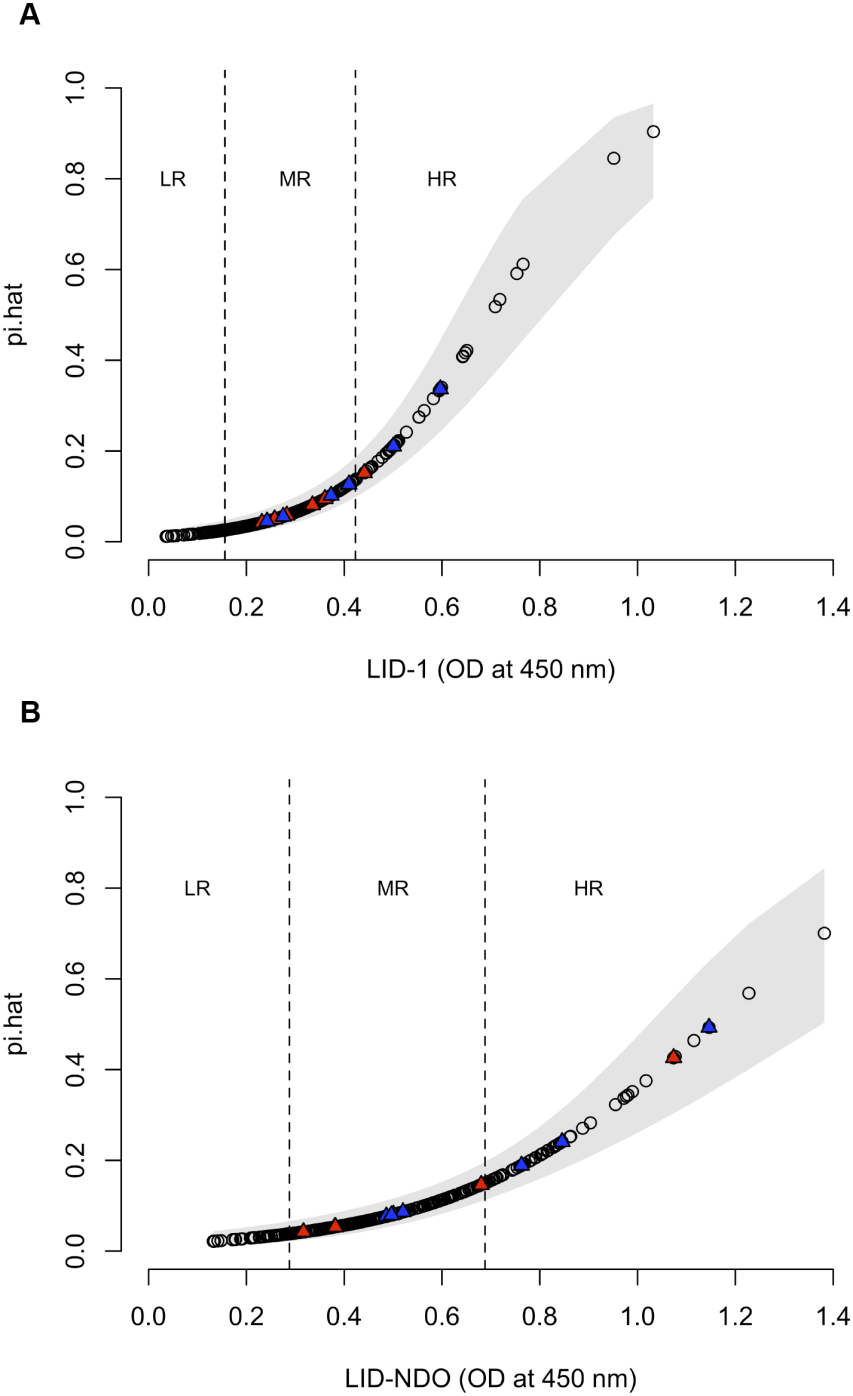
Probability estimates (pi.hat) of asymptomatic infected HHC developing leprosy according to antibody levels. A total of 332 household contacts living in the hyperendemic area of Mossoró, Rio Grande do Norte, Brazil, were analyzed based on anti-LID-1 (A) and anti-LID-NDO (B) antibody levels. Blue and red triangles represent contacts that developed PB and MB leprosy, respectively. The cut-off values for scenarios 2 and 3 (dashed lines) were used to group individuals by risk (LR: low risk; MR: moderate risk; HR: high risk).

For this analysis, we included household contacts (n = 332) and leprosy cases (n = 50, encompassing 8 PB and 42 MB) from the hyperendemic region of Mossoró. Fig 8A and 8B show the predicted probabilities (pi.hat) as a function of LID-1 and LID-NDO values, respectively. By computing the mean probability among all contacts, we projected a new case detection rate (*i*.*e*. incidence) of 8.3% based on LID-1 ELISA (Fig 8A) and 10.4% with LID-NDO ELISA (Fig 8B). The cut-off values for scenarios 2 (to diagnose MB cases in a high-risk population) and 3 (to identify asymptomatic-exposed individuals) were used to group individuals by risk (LR: low risk; MR: moderate risk; HR: high risk). The incidence rate for each group according to the antigen tested is in Table 3. The relative risk (RR) using LR as reference of a HHC classified as HR to develop disease was at least 7.7 times greater than those classified as LR. Interestingly, no HHC that developed leprosy in the longitudinal analysis was on the LR group (Fig 8A and 8B).

**Table 3:** HHC groups of risk based on antibody levels.

Group based on antibody levels	LID-1		LID-NDO	
	Incidence rate	RR	Incidence rate	RR
LR	0.021	1.0	0.032	1.0
MR	0.059	2.8	0.080	2.5
HR	0.302	14.1	0.246	7.7
Overall	0.083	-	0.104	-

RR: relative risk.

## Discussion

Despite the significant decrease in the global prevalence of leprosy, since the introduction of MDT, there are still many regions where the detection of new leprosy cases remains high [29]. Overall, active case finding studies indicate that the true prevalence of leprosy is still probably grossly underestimated. Diagnostic limitations hinder large-scale control programs aimed at the eventual elimination of this disease. In this sense, studies have shown that serologic tests may contribute to early diagnosis, even before the appearance of lesions [18;30;31]. In this study, we evaluated the presence of antibodies against the recombinant fusion protein LID-1 and the glycoprotein conjugate LID-NDO, in groups of individuals with leprosy or with prolonged exposure to *M*. *leprae*. Our data indicate that ELISA detecting antibodies against LID-1 and LID-NDO had high sensitivity and specificity to aid the diagnosis of leprosy and to identify people with asymptomatic *M*. *leprae* infection prior to lesion development.

We observed the highest antibody responses in MB with the clinical forms LL/BL and the magnitude of response declined throughout the clinical spectrum, with the lowest values observed in TT patients. Accordingly, the titers of specific antibodies correlated positively with bacillary indexes of patients [23;32]. These data confirm that these serum antibody responses can closely reflect infection levels and indicate that this could be a simpler, less invasive technique than skin slit smears to estimate *M*. *leprae* bacterial burden. Importantly, the serological tests were more sensitivity than bacilloscopy. Thus, these tests can certainly contribute to the accurate leprosy diagnosis, especially in areas where histopathological exams are not available.

Due to a shared environment, and likely an increased exposure, it is recognized that HHC represent a higher risk group for the development of leprosy. This is especially true when contact is with a heavily infected MB case [33–35]. As indicated by circulating antibody levels, our study suggests that a large proportion of the HHC evaluated were likely to have been previously exposed to *M*. *leprae* infection. This was expected since the participants were recruited in a hyperendemic area of Rio Grande do Norte [26]. We found that HHC of MB cases had, on average, higher levels of anti-LID-NDO than HHC of PB cases. Interestingly, HHC of MB patients also presented with higher level of antibodies than PB patients. This finding is in accordance with the results obtained for LID-1 and LID-NDO in another population in Brazil [23]. A possible explanation for this is that PB leprosy case mount an effective cellular immune response to control bacterial replication, and, consequently, mitigating antibody responses [8;9]. On the other hand, as our longitudinal study indicates, household contacts of MB patients may develop an antibody response, while either resolving infection or the possibility of subsequently developing clinical disease. In *Leishmania infantum* infection which can also have a spectrum of outcomes as *M*. *leprae*, an inverse correlation between antibody and cellular immune responses was observed [36].

Study of the seropositivity rates against PGL-I in Minas Gerais, Brazil, detected responses among HHC at a rate of 10.4% [34]. In our analysis, considering the cut-off used to define HHC positive for MB diagnosis, we found that seropositivity rates among the HHC of 11.9% and 17.8% for LID-1 and LID-NDO, respectively. This is a relatively high percentage, likely related to the high endemicity within the area from where this population was recruited [27]. In fact, when we considered the cut-off based on EC, the majority of HHC recruited into our study presented with antibody levels indicative of asymptomatic *M*. *leprae* infection, emphasizing the potential for leprosy to emerge and become a greater problem in the region.

Most people exposed to *M*. *leprae* develop a protective immune response and do not develop leprosy [37]. However, especially in hyperendemic areas, it is critical to develop strategies to control the transmission of *M*. *leprae*. Although not yet formally shown for LID-NDO, a previous study showed the application of LID-1 for the detection of cases up to a year before the recognition of lesions [18]. In our longitudinal study, among the 332 HHC recruited between 2006 and 2008 in the hyperendemic area of Mossoró, 3.6% were reported as developing leprosy at a later date. Interestingly, some of these HHC presented with positive serum antibody responses against LID-1 and/or LID-NDO years before the onset of clinical signs of leprosy. This suggests that LID-1 or LID-NDO could be used as a tool for early identification of *M*. *leprae*-infected individuals to increase vigilance to expedite the diagnosis of leprosy.

We compared the results obtained in our cohort with a model to predict leprosy development for HHC based on antibody levels. The probability of a HHC with positive serology to develop leprosy was 8.3% for LID-1 or 10.4% for LID-NDO. The difference between the value estimated by the model and the one observed in the cohort (3.6%) may arise due to passive case detection in the area, since studies show that the actual incidence of the disease can be much higher during active case finding [27;38]. Taken together, at a minimum, this indicates that these antigens could have helped in monitoring these individuals to provide the early diagnosis of new cases.

According to the levels of specific antibodies presented by HHC, we were able to define three groups based on risk of developing leprosy. Interestingly, when we investigated our cohort for HHC who developed leprosy we observed that they arose from the moderate and high risk groups, but not from the low risk. This reinforces the importance of conducting serological surveys in populations considered at risk (*i*.*e*. HHC) in endemic regions.

In summary, our data indicate that both LID-1 and LID-NDO ELISA represent important tools for the diagnosis of leprosy. Our data also indicate that these ELISA can be used to estimate the bacterial load of patients. In a leprosy endemic country like Brazil it is essential that new auxiliary techniques become available for disease control in various states and municipalities. Serological tests that do not require significant labor and can detect asymptomatic *M*. *leprae* infection may contribute to the control and eradication of leprosy.

## Supporting Information

S1 Table Comparison of LID-1 OD results between the leprosy spectrum. (PDF)

S2 Table Comparison of LID-NDO OD results between the leprosy spectrum. (PDF)

S3 Table ELISA tests sensitivity and specificity for leprosy diagnosis considering the mean OD of the endemic control plus three standard deviation. (PDF)

S1 Checklist STARD checklist. (DOCX)
