# Supplementary material for: Identifying Leprosy and Those at Risk of Developing Leprosy by Detection of Antibodies against LID-1 and LID-NDO
Source: PLoS Negl Trop Dis. 2016 Sep 22;10(9):e0004934. doi: 10.1371/journal.pntd.0004934 (PMC5033353; doi:10.1371/journal.pntd.0004934)
Supplement: S1 Table — (PDF) [file pntd.0004934.s001.pdf]

S1 Table. Comparison of LID-1 OD results between the leprosy spectrum.

Groups	Mean difference	Adjusted p-value*
BT-TT	0.353	0.008
BB-TT	0.297	0.297
BL-TT	1.116	< 0.001
LL-TT	1.087	< 0.001
BB-BT	-0.056	0.996
BL-BT	0.763	< 0.001
LL-BT	0.735	< 0.001
BL-BB	0.819	< 0.001
LL-BB	0.790	< 0.001
LL-BL	-0.028	1

* p-values adjusted for multiple comparisons (Tukey’s test)

S2 Table. Comparison of LID-NDO OD results between the leprosy spectrum.

Groups	Mean difference	Adjusted p-value*
BT-TT	0.310	0.059
BB-TT	0.443	0.082
BL-TT	1.235	< 0.001
LL-TT	1.084	< 0.001
BB-BT	0.133	0.939
BL-BT	0.925	< 0.001
LL-BT	0.774	< 0.001
BL-BB	0.792	0.001
LL-BB	0.641	0.013
LL-BL	-0.151	0.858

* p-values adjusted for multiple comparisons (Tukey's test)
