# Supplementary material for: Identifying Leprosy and Those at Risk of Developing Leprosy by Detection of Antibodies against LID-1 and LID-NDO
Source: PLoS Negl Trop Dis. 2016 Sep 22;10(9):e0004934. doi: 10.1371/journal.pntd.0004934 (PMC5033353; doi:10.1371/journal.pntd.0004934)
Supplement: S3 Table — (PDF) [file pntd.0004934.s003.pdf]

S3 Table. ELISA tests sensitivity and specificity for leprosy diagnosis considering the mean OD the endemic control + 3 standard deviations

Test	EC			PB			MB			PB/MB	
	n	Specificity		n	Sensitivity		n	Sensitivity		n	Sensitivity
**LID-1**											
Negative	97	99% (97/98)		27	16% (5/32)		8	88% (58/66)		35	64% (63/98)
Positive#	1			5			58			63	
Total	98			32			66			98	
**LID-NDO**											
Negative	96	98% (96/98)		30	6% (2/32)		23	65% (43/66)		53	46% (45/98)
Positive#	2			2			43			45	
Total	98			32			66			98	

Individuals were defined as positive if OD value > cut-off.
Cut-off = *mean + 3xSD*, calculated using OD values for endemic control group (EC).
